# Unsupervised domain adaptation methods for cross-species transfer of regulatory code signals

**DOI:** 10.3389/fdata.2023.1140663

**Published:** 2023-03-30

**Authors:** Pavel Latyshev, Fedor Pavlov, Alan Herbert, Maria Poptsova

**Affiliations:** ^1^Laboratory of Bioinformatics, Faculty of Computer Science, HSE University, Moscow, Russia; ^2^InsideOutBio, Charlestown, MA, United States

**Keywords:** transfer learning, domain adaptation, domain adversarial networks, versatile domain adaptation, Minimum Class Confusion, histone marks, transcription factors

## Abstract

Due to advances in NGS technologies whole-genome maps of various functional genomic elements were generated for a dozen of species, however experiments are still expensive and are not available for many species of interest. Deep learning methods became the state-of-the-art computational methods to analyze the available data, but the focus is often only on the species studied. Here we take advantage of the progresses in Transfer Learning in the area of Unsupervised Domain Adaption (UDA) and tested nine UDA methods for prediction of regulatory code signals for genomes of other species. We tested each deep learning implementation by training the model on experimental data from one species, then refined the model using the genome sequence of the target species for which we wanted to make predictions. Among nine tested domain adaptation architectures non-adversarial methods Minimum Class Confusion (MCC) and Deep Adaptation Network (DAN) significantly outperformed others. Conditional Domain Adversarial Network (CDAN) appeared as the third best architecture. Here we provide an empirical assessment of each approach using real world data. The different approaches were tested on ChIP-seq data for transcription factor binding sites and histone marks on human and mouse genomes, but is generalizable to any cross-species transfer of interest. We tested the efficiency of each method using species where experimental data was available for both. The results allows us to assess how well each implementation will work for species for which only limited experimental data is available and will inform the design of future experiments in these understudied organisms. Overall, our results proved the validity of UDA methods for generation of missing experimental data for histone marks and transcription factor binding sites in various genomes and highlights how robust the various approaches are to data that is incomplete, noisy and susceptible to analytic bias.

## 1. Introduction

**Figure 1:**
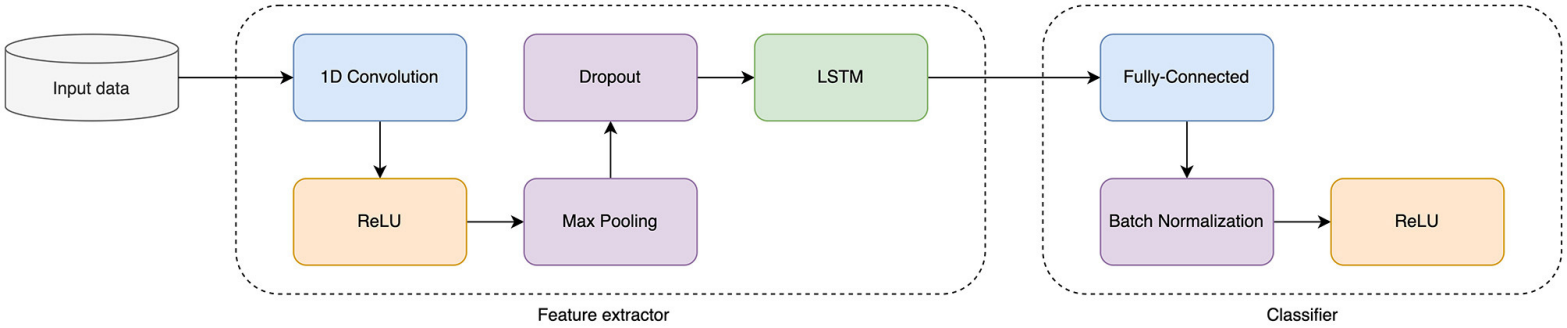
General schema for domain adaptation models. In contrast to the standard deep learning methods, DA approaches use two separate sets of training data: source domain data both with genomic regions and corresponding labels, and target domain unlabeled region dataset. The second dataset allows the transfer learning mechanism to work, as after the model training stage the learned features in the feature extractor would contain information that is specific for both source and target domains.

Domain Adaptation (DA) methods were designed for the task to transfer knowledge from a labeled source domain to an unlabeled target domain. Unsupervised DA (UDA) is an approach when source domain and target domain share the same label set. Most of the UDA approaches can be roughly divided into three types: divergence-based, adversarial-based and reconstruction-based methods (Jiang et al., 2022). The divergence-based methods try to minimize the distances between distributions of the source and target domains and usually minimize two losses during training—classification loss and divergence-based loss. Adversarial-based methods are based on the same approach used in Generative Adversarial Networks (GANs) where generator serves as a feature extractor and discriminator learns to distinguish between source and target domains. In this case optimization is based on classification loss and discriminator loss. In reconstruction-based domain adaptation, the target features are translated into source features (for example, with encoder-decoder modules) and then a discriminator is trained to distinguish between the generated and source domain. Recently a completely different approach was proposed that employs a novel minimum class confusion (MCC) loss-function (Jin et al., 2020). Since MCC loss function can be used with any DA method, the approach was named as Versatile Domain Adaption (VDA). In this paper we focus only on three types of DA models—divergence-based, adversarial-based and versatile DA with MCC and test their robustness of their predictions with real world data. The general schema of DA approach is presented in Figure 1.

Transfer learning methods have been already successfully applied to solve various tasks in genomics. Transfer learning was used for cancer classification based on gene expression data where for tumor classification the method transferred feature representation from other tumors (Sevakula et al., 2018). Transfer learning was helpful in denoising scRNA-seq data using Bayesian hierarchical model coupled with a pretrainable deep autoencoder (Wang et al., 2019). Domain adversarial neural network was used for prediction of enhancer promoter interactions in a cell line of interest by learning features from enhancers and promoters shared among all cell lines (Jing et al., 2020). Few-shot learning and meta-transfer learning were explored on large data sets from TCGA and GTEx and the approach helped in addressing heterogeneity, batch effects and other forms of bias in omics data analyses (Park et al., 2021). Functional microexons were predicted when prior knowledge from microindels was transferred to the final model (Cheng et al., 2021). In single cell sequencing, transfer learning was successfully applied to transfer cell annotations from labeled data sets from one experiment to another unlabeled data sets (Kimmel and Kelley, 2021). Another single cell applications is an integration of multiple single-cell datasets across samples employing algorithm based on domain-adversarial and variational approximations (Hu et al., 2022). Validity of domain adaptation approach based on Domain Adversarial Neural Network (DANN) was demonstrated for to the task of cross-species TF binding prediction (Cochran et al., 2022).

Whole-genome maps for different genomic elements have been continuously generated by the Encode project (Luo et al., 2020). However, many experiments for a particular species are often absent. Here we take advantage of the transfer learning methods and test the unsupervised domain adaptation methods to generate missing experiment labels based on the knowledge gained from experimental data from another species. In the proposed approach, a good performing deep learning model is trained on the genome of one species and then it is applied to another species after additional training with the targeted genomic sequence. Each method currently available for performing this task is quite computationally expensive. In this current work we compare each implementation using the same real world datasets to provide a benchmark for others to use and for future improvements to the underlying algorithms.

**Figure 2:**
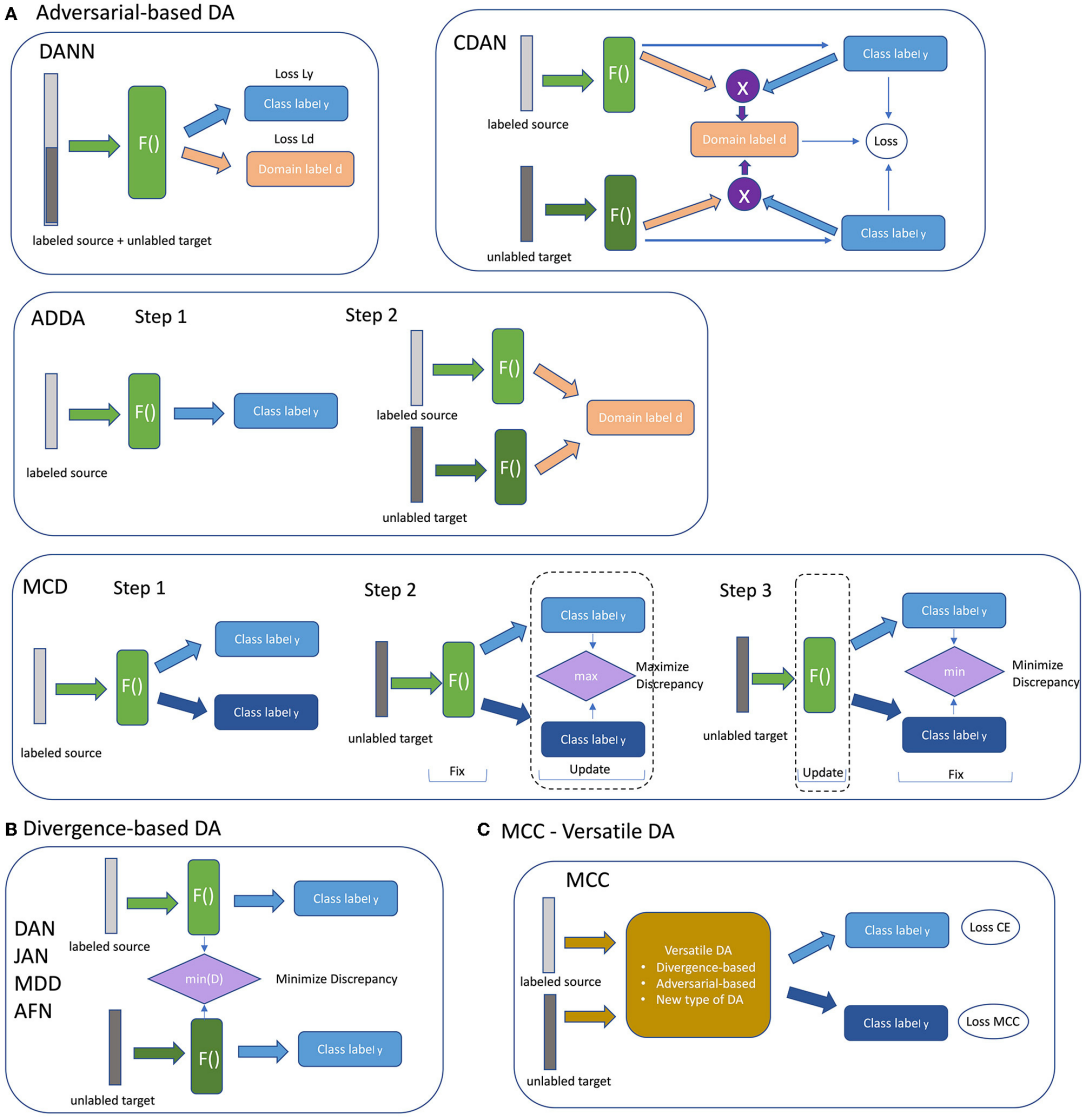
Schematic representation of different types of DA models. **(A)** Adversarial-based DA models. **(B)** Divergence-based DA models. **(C)** MCC with Versatile DA models.

We tested nine domain adaptation methods: Domain Adversarial Neural Network (DANN) (Ganin et al., 2016), Deep Adaptation Network (DAN) (Long et al., 2015), Joint Adaptation Network (JAN) (Long et al., 2017), Adversarial Discriminative Domain Adaptation (ADDA) (Tzeng et al., 2017), Conditional Domain Adversarial Network (CDAN) (Long et al., 2018), Maximum Classifier Discrepancy (MCD) (Saito et al., 2018), Adaptive Feature Norm (AFN) (Xu et al., 2019), Margin Disparity Discrepancy (MDD) (Zhang et al., 2019), and Minimum Class Confusion (MCC) (Jin et al., 2020). DANN, CDAN, ADDA, MCD are adversarial-based approaches; DAN, JAN, MDD, AFN are divergence-based approaches; and MCC is a versatile DA method. All the methods differ from each by the way labeled source and unlabeled target data are supplied during the training. Schematically all the approaches are presented in Figure 2. Adversarial-based DA methods differ from each other by the way the adversarial training is organized. Some are trained as a whole, and some are sequentially, such as ADDA and MCD (Figure 2A). Divergence-based method are more similar to each other and differ only in methods they minimize divergence of feature representations (Figure 2B). The difference between each approach is reflected in the specific details of how each is implemented.

Domain adversarial learning methods are based on using adversarial neural networks inspired by GAN (Generative Adversarial Network). DANN (Domain Adversarial Neural Network) consists of three blocks. The first block is a feature generator that extracts a hidden representation of data. The second block is a classifier that makes class predictions using the hidden representation. The last block is a discriminator that predicts the domain of the data. The model is trained on labeled data from the source domain and unlabeled data from the target domain in a way that the discriminator tries to distinguish between the source domain and target domain, the feature extractor tries to make it indistinguishable, and the classifier block tries to predict the label of an object. As a result, a model from this class is trained to extract the common features for both target and source domains. Domain transfer works better when predictions are made based on features that cannot discriminate between the source and target domains.

The difference between DANN and ADDA (Adversarial Discriminative Domain Adaptation) is in the training process. ADDA, unlike DANN, splits the optimization of the discriminator and the feature extractor into two parts. Representation learning is done at the first step from the source domain. At the second step, called adversarial adaptation, training is performed in an adversarial manner when discriminator cannot reliably predict domain labels of the encoded source and target samples.

CDANs are inspired by conditional GANs and implement conditioning of the cross- covariance between feature representations and entropy conditioning of the uncertainty of classifier predictions.

MCD (Maximum Classifier Discrepancy) is based on two players to align distributions: feature generator and domain classifier are trained in adversarial manner. At one step the model is trained to maximize the discrepancy between the source classifier predictions and target classifier predictions to detect the samples that are far from the source domain. At the next step a feature generator learns to generate target features to minimize the discrepancy (Figure 2A).

Divergence-based methods have the common general structure and differ in a method used to minimize discrepancy between feature distributions of source and target domains (Figure 2B). MDD (Margin Disparity Discrepancy) minimizes a new proposed metric, called the disparity discrepancy metric, to measure the discrepancy between source and target classifiers. In the original paper with AFN (Adaptive Feature Norm) method (Xu et al., 2019), the authors empirically discovered that the quality degradation on a target domain roots from much smaller feature norms of the classifier's feature norms on target domains with respect to the feature norms of the source domain. This method progressively adapts the feature norms of the two domains to a larger range of values to improve transfer gains.

JAN and DAN are methods from the statistics matching class. These methods adapt distributions of features in models. JAN (Joint Adaptation Network) aligns the joint distributions of multiple domain-specific layers across domains based on a joint maximum mean discrepancy criterion which measures the Hilbert-Schmidt norm between kernel mean embedding of empirical joint distributions of source and target data (Long et al., 2017). DAN (Deep Adaptation Network) aligns marginal distributions of activations in multiple domain-specific layers. This is achieved by mapping of embeddings of all the task-specific layers to kernel Hilbert space. Thus, the mean embeddings of different domain distributions can be explicitly matched. Kernel choice can be optimized to select the one that reduces domain discrepancy (Long et al., 2015).

MCC (Minimum Class Confusion) is a non-adversarial Versatile Domain Adaptation method with a novel loss function based on entropy minimization methods. This type of method tries to minimize entropy on unlabeled target class objects to increase the confidence of predictions. The method does not explicitly align domains leading to a faster convergence speed. The main idea behind the method stems from the fact that classifier predictions initially confuse the correct and ambiguous classes, and it tries to minimize the confusion. In MCC realization feature extractor is shared between source labeled and target unlabeled data, and MCC loss function is defined on the class prediction of the target data.

In the original publications all these DA methods were tested on image datasets that can be precisely measured, allowing an evaluation of how noise, missing data, spatial orientation and other confounder variables affect predictions. It is of interest to test applicability of those methods to experimental molecular biology data sets that are incomplete, noisy, and subject to other biases. However, experiments are expensive and lacking for many species of interest, thus understanding the potential of transfer learning in genomics is of practical importance to guide future experiments and to improve our knowledge of how species differ. We tested the described above nine methods on various experimental ChIP-seq data for histone marks and transcription factor binding sites for cross-species predictions between human and mouse genomes.

## 2. Results

### 2.1. Deep learning model to predict transcription factor binding sites and histone mark signals

**Figure 3:**
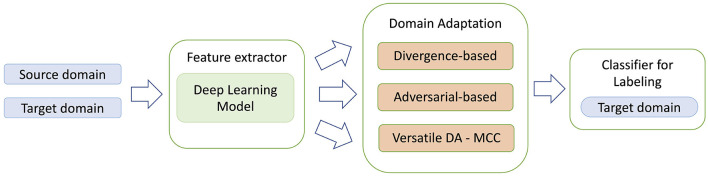
Schema of a Baseline Deep Learning Model for prediction of TF and HM signals.

First, we constructed a deep learning model to predict transcription factor (TF) binding sites (TFBS) and histone mark (HM) positions in the source genome from DNA sequence. DNA regions were one-hot encoded into binary matrices. For the classifier we chose a hybrid CNN and LSTM network as it showed the best performance over single CNN or single LSTM networks on our TFBS and HM data sets. The schema of the selected deep learning model is presented in Figure 3.

For all tested data sets we first trained the source model and with the trained source model we made predictions on the target genomes.

### 2.2. Comparison of unsupervised domain adaptation methods

We tested nine UDA methods from three types of DA approaches: adversarial-based methods, divergence-based methods, and versatile domain adaption approach based on MCC loss function. The general schema for domain adaptation models is presented in Figure 1. In this scheme TFBS or HM sequence representations are learned with a hybrid CNN+LSTM network and then the downstream task is solved either by minimizing the distance between two domain distributions, or in minimax adversarial manner, or by minimizing the class confusion metric.

We tested 9 UDA methods on 10 histone marks (H2B, H3K23ac, H3K36ac, H3K36me1, H3K79me1, H3K27ac, H3K4me1, H3K4me2, H3K4me3, and H3K9me3) and 4 transcription factors (CTCF, RAD21, SPI1 and TBX21) on blood, kidney, lung, liver, breast, prostate, pancreas, neural tissues, and stem cells totaling 71 experimental data sets for both genomes (see Methods for the full list of tissue and cell types). We chose those experimental data sets for which data was available for both human and mouse genome. Some histone marks are not frequent so that samples from one tissue type do not contain enough data needed for training. In this case we combined different tissue types in one data set.

**Figure 4:**
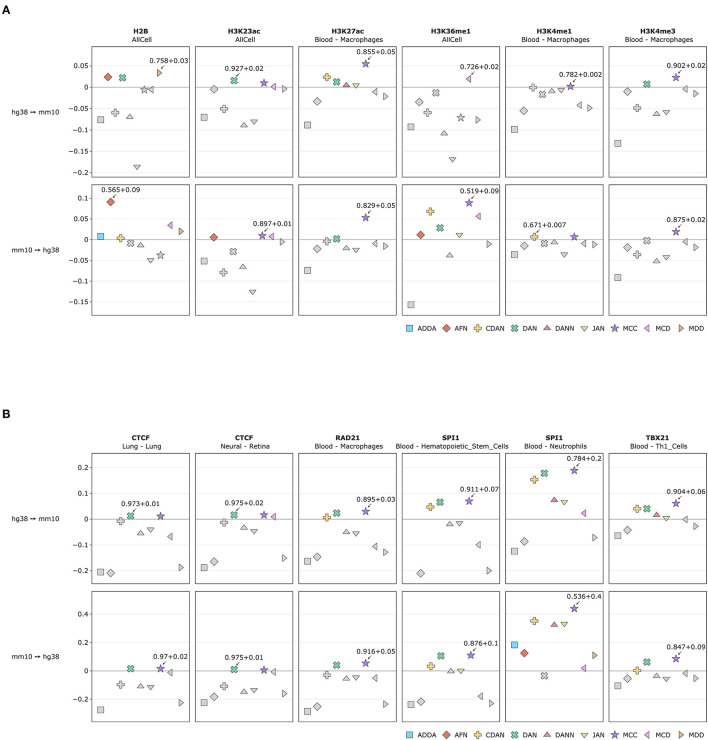
Comparative performance (ROC AUC) of Domain Adaptation methods for cross-species prediction of **(A)** 6 selected histone marks and **(B)** 6 selected transcription factors. Y axis depicts difference between DA performance metrics (ROC AUC) with respect to the baseline model, i.e., model trained on the source genome and applied to the target genome directly without DA approach (see comparison based on PR AUC and accuracy in Supplementary Figure 1). The best performing DA model is marked with an arrow with the corresponding metrics of the baseline model plus a gain in performance achieved by the DA model. See full list of experiments in Supplementary Table 1.

**Figure 5:**
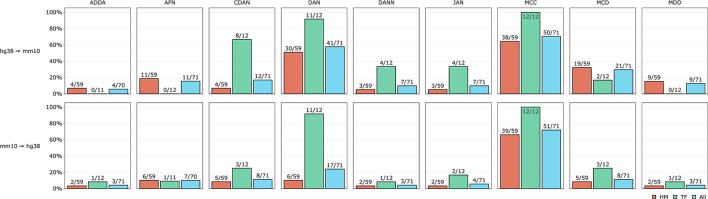
Comparative performance of 9 DA models tested on TFs and HMs human-mouse and mouse-human cross-species predictions. Y axis depicts percentage of DA experiments when at least 1 out of 9 DA models worked better than the baseline model. Absolute numbers are given next to the corresponding bars.

**Figure 6:**
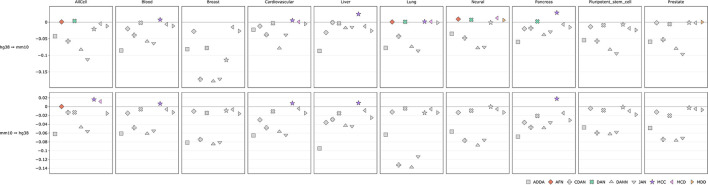
Comparison of DA model performance (ROC AUC) across tissues for histone marks (see comparison based on PR AUC and accuracy in Supplementary Figure 4).

**Figure 7:**
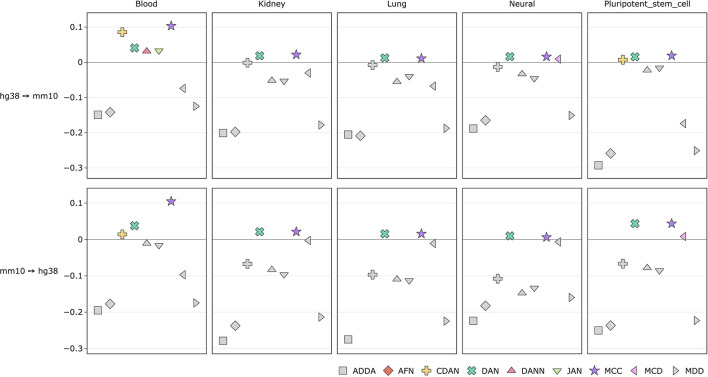
Comparison of DA model performance (ROC AUC) across tissues for transcription factors (see comparison based on PR AUC and accuracy in Supplementary Figure 5).

We first trained the deep learning model on the source genome, either human or mouse, and tried to predict regulatory signals on the target genome without transfer learning (referred to as the baseline model). Then we run nine DA models and compared the performance of each model (referred to as the TL model) with the performance of the baseline model based on accuracy, PR AUC and ROC AUC. In total we ran 1,420 models: 1 baseline model and 9 DA TL models for 59 HM and 12 TF ChIP-seq peak sets for human → mouse and mouse → human transfer learning experiments. The summary results are presented in Figures 4–7 and Supplementary Table 1.

The comparison of DA performance metrics (ROC AUC) with respect to the baseline model for selected 6 HMs are presented in Figure 4A; comparison based on PR AUC and accuracy are presented in Supplementary Figure 1. For 3 histone marks—H2B, H3K23ac and H3K36me1—experiments were combined in one set from all available tissue types. The figure shows the difference between the baseline model and the TL model. For other 3 histone marks—H3K27ac, H3K4me1 and H3K4me3—the experiments were taken for macrophages and blood tissue. The absolute values for all DA model metrics along the baseline model for different HMs and different cells and tissues are given in Supplementary Table 1.

The comparison of DA performance metrics for the TL model (ROC AUC) with respect to the baseline model for selected 6 TFs are presented in Figure 4B; comparison based on PR AUC and accuracy are presented in Supplementary Figure 2. 6 TF ChIP-seq experiments include CTCF for lung and neural tissue, RAD21 for macrophages, SPI1 hematopoietic stem cells and neutrophils, and TBX21 for T-helper cells. The absolute values for all DA model metrics along the source model for all TF experiments are given in Supplementary Table 1.

To estimate the validity of DA approach we counted how many times each DA model worked better than the baseline model separately for HMs and TFs and for all experiments jointly. The comparison results are presented in Figure 5. We can see that non-adversarial method MCC is an absolute winner for both TFs and HMs when transferring representations from human to mouse and from mouse to human genomes. Divergence-based approach DAN appeared to be the second-best method for TFs for both human and mouse genomes but for HMs mostly when transferring knowledge from human to mouse. Among adversarial methods, we observed that conditional domain adaptation approach CDAN worked for some sets of histone marks when transferring annotations from human to mouse (but not vice versa) for some HMs. The same is true but to a lesser extent for adversarial-based method MCD. Two other adversarial models—DANN and ADDA—did not perform better than the baseline model for most of the experiments. The results of DA model comparison based on PR AUC and accuracy are given in Supplementary Figure 3.

### 2.3. Tissue-specific vs. combined regulatory signal prediction

In case of histone marks, we tested UDA methods for tissue-specific and combined HM signals and found that combined approach works better for sparse HM signals (for example, H3K36me1 and H3K23ac) as compared to the dense H3K27ac. For tissue-specific predictions the baseline model applied directly to the target genome without domain adaptation performs with a good prediction power so that DA approach brings relatively little improvement. However, in cases when the baseline model initially does not perform well on the target genome, DA methods can improve predictions by up to 9% of ROC AUC. For example, for H3K4me1 in liver hepatocytes or for H3K36me1 for the combined data set from all available tissues and cell types the increase in model performance is 8.74 and 8.86% correspondingly (Figure 4A, Supplementary Table 1).

TFBS signals are more tissue specific and thus we did not combine TFBS from different tissues in one set. When TFBS are compared to HMs, we observe even larger positive effect of DA with an improvement in model predictions power on average by up to 29% (as in the case for SPI1 signal in dendritic cells and neutrophils) when comparing to the baseline model without DA (Figure 4B, Supplementary Table 1).

We also measured average performance gains for each DA method across each tissue both for histone marks and transcription factors (Figures 6, 7, Supplementary Figures 4, 5). For histone marks (Figure 6, Supplementary Figure 4) in case of human-to-mouse conversion we can see small score-wise improvements in every tissue of interest except for the breast and pluripotent stem cells. The highest gains of around ~3% are achieved for the MCC method. Interestingly, in all cases except for the “all cells” for mouse-to-human conversion we do not observe DA method performance improvements except for the MCC, which scores higher on average in blood, cardiovascular, liver and pancreas cells. ADDA, CDAN, DANN, and JAN show no average performance gains across the observed tissues.

The score improvements are more significant when measured across transcription factor tissues (Figure 7, Supplementary Figure 5). For both transfer learning tasks we can observe up-to 11% ROC AUC score gains. In case of mouse-to-human conversion the DAN and MCC methods constantly perform better than the baseline source-only model. ADDA and MDD show no average performance gains with ADDA being the least effective one.

Overall direct cross-species predictions, with or without DA, work better for TFBS than for HM indicating the more conserved nature of sequences bearing TF binding signals.

## 3. Discussion

The general schema of transfer learning models can be represented as a two-stage process during which a model first is pretrained for gaining the transferable knowledge (feature representations) and then adapted to the downstream task to predict labels in the target domain (Jiang et al., 2022). When the target domain has unlabeled data, then the source data with the same labels but different distribution is used to improve domain adaptation (Ganin and Lempitsky, 2015).

Our task of transferring signals of genomic regulatory code from one species to the other corresponds to the case when a source domain with labeled data (experimental data from one genome) is used to improve the prediction quality of a classifier of a target domain with unlabeled data (regulatory code signals in another genome). Representations of sequences for TF binding sites or HM locations in human and mouse genomes come from different but similar distributions. The goal is to select features that cannot discriminate between the source and target domains, i.e., invariant features of the genomic regulatory code.

The initially trained deep learning model (here, hybrid CNN-LSTM but any deep learning model could be used) provides feature representations of both source and target genomes. The distributions of these sequence-based representations are close but different due to species-specific differences. The task of domain adaptation approaches is then to reduce the domain dissimilarity. This goal is achieved either by divergence-based methods or adversarial-based methods. Divergence-based methods try to make domain and source feature distribution more similar based on some divergence criteria. Adversarial-based methods work for extracting invariant features for both domains due to the addition of a module where domain discriminator is trained to distinguish source and target features. Our results showed that among four adversarial-based methods, only conditional domain adversarial network (CDAN) showed reasonably good performance, being the third-best after the MCC and divergence-based method DAN.

CDAN approach was borrowed from conditional GANs. In case of adversarial domain adaptation models, the conditioned module is a domain discriminator. The domain discriminator is conditioned on the cross-covariance of domain-specific feature representations and classifier predictions. For our data, CDAN worked very well for SPI1 data sets with a significant (up to 29 %) increase in model performance and it also worked, though not that noticeable for CTCF (4 out of 6 experiments). Another adversarial method MCD showed a small surplus by <0.5 mostly for HM and mostly for human to mouse label transfer.

MCC is neither adversarial nor divergence-based method. It introduces a novel loss function—Minimum Class Confusion (MCC). MCC is defined on the target label predictions produced by the source classifier on the target data. With this definition MCC can be applied to both adversarial and divergence-based methods, hence the name: Versatile Domain Adaption (VDA). The VDA method does not explicitly align domains and thus has faster convergence speed. MCC showed the highest performance for both TFs and HMs and in both knowledge transfer directions: from human to mouse and from mouse to human. In the original MCC publication for a UDA task based on image classification, the method performance in some instances compared to that of CDAN, similar to what we observe for our genomic sequence data sets where CDAN is the second-best method. Also, in the original publication, the authors showed that MCC can be used as a general regularizer for other DA methods, especially for CDAN and AFN.

In the original publication MCC method for UDA tasks was trained on standard image data sets: VisDA-2017 and Office-31. MCC outperforms all current DANN, DAN JAN, CDAN, AFN, MDD (Jin et al., 2020). When MCC was used as regularizer, it yielded larger improvements when compared to entropy minimization and batch spectral penalization to adversarial-based DA methods such as DANN, CDAN and divergence-based approach AFN. The second and third best models along with MCC in its original publication are CDAN and AFN for VisDA-2017 and CDAN and MDD for Office-31 data set. While CDAN is also in the top-3 models for genomics ChiP-seq data, the second-best—DAN– second before last for image data sets.

DAN is a divergence-based method that employs the multiple kernel variant of maximum mean discrepancies (MK-MMD). DAN is represented as a multilayer architecture for learning transferable features. Features are transitioned from general to specific when the first three layers are general and frozen, then the fourth and fifth layers are slightly less transferable, hence these layers are learned *via* fine-tuning, and then remaining fully connected layers are adapted with MK-MDD and are not transferable and aimed at fitting to specific tasks [see Figure 1 in Long et al., 2015]. Obviously, this method better finds features invariant for both domains, however the theoretical grounds of the observed phenomenon remain out of the scope of the presented study.

Overall, when comparing patterns of DA model performance, the results are qualitatively the same for all TFs and all types of tissues, with MCC and DAN being the two best models when the domain adaptation approach provides a surplus for model performance compared to the direct application of the source model predicting target labels. Patterns of HMs are less obvious, but MCC remains an absolute winner.

Relatively high cross-species deep learning model performance confirms the presence of evolutionary conserved sequence determinants. Machine learning approach based on SVM and CNNs were tested for cross-species predictions of enhancers in mammalian species (Chen et al., 2018) and even without transfer learning, the findings confirmed presence of evolutionary conserved signals. The DANN approach was recently tested on cross-species prediction of transcription factor binding in human and mouse and demonstrated the viability of the approach (Cochran et al., 2022). The authors explored effect of species-specific transposon repeats on DA methods when they introduce noise in the sequences around detectable signals. We obtained results that are consistent with the results obtained for TFs in cross-species mouse-human and human-mouse predictions for one type of DA models—DANN. We extended the approach up to nine models and also enlarged it with histone mark annotations.

The results of our tests showed that non-adversarial methods work better for this specific genomic task, even without additional filtering for obvious species-specific sequences such as human Alu and mouse B1 transposons. Overall, the DA approach already shows sufficient quality and can be of practical use for cross-species transfer for human and mouse genomes. With DA models researchers can generate predictions about gene regulation in new species to guide experimentation, produce results and reiterate until we have a good enough model to explain the difference, if any, between the source and target species.

Another direction of research is to explore invariant sequence features of regulatory code across evolutionary divergent species. Those invariant features will correspond to evolutionary-conserved DNA motifs. With DA approach it is possible not only extract invariant sequence features but also select subset of divergent species- and/or tissue-specific. For that task use of CNN architecture will be more practical since it is possible to convert active filters to DNA motifs.

The study presented here provides a practical guide which models work better, and how to choose the right DA approach to transfer experimental results from genome of one species to another.

## 4. Conclusion

Even if the cost of sequencing significantly decreases, it is unfeasible to perform all needed experiments for location of numerous regulatory elements such as histone marks and transcription factor binding sites for all tissue types and all species of interest. We demonstrate that the knowledge of regulatory code in one genome can be efficiently transferred to the other evolutionary close genomes such as human and mouse. We tested and compared performance of different types of DA models and found that a novel type of DA methods based on the MCC loss function works better than adversarial and divergence-based DA models for our genomic regulatory signal transfer tasks. Partially this is due to the noise, incomplete and biased nature of high-throughput molecular biology experiments, though MCC also outperformed other DA methods on image data. In our further research we plan to extend testing of DA methods on evolutionary more divergent species to understand the limits of cross-species regulatory code transfer. Entropy-based methods to reduce mislabeling of target data show great promise.

## 5. Methods

### 5.1. Data sets

Complete genomic sequences for mouse (mm10) and human (hg38) assembly versions were downloaded from the UCSC genome browser (Lee et al., 2022). Histone mark and transcription factor data sets for different tissue types were downloaded from ChIP-atlas database (Zou et al., 2022). ChIP-seq experiments for some histone marks do not contain enough data for training. For these experiments we combined datasets from different tissues into one. The size of each data set in human and mouse genome is provided in Supplementary Table 2.

#### 5.1.2. Histone mark data

H2B all, H3K23ac all, H3K36ac all, H3K79me1 all, H3K27ac: Blood (B cells, CD4+ T cells, CD8+ T cells, Dendritic Cells, Erythroblasts, Hematopoietic Stem Cells, Macrophages), Breast (Mammary glands), Cardiovascular (Heart Ventricles), H3K27ac: Liver (Hepatocytes), Neural (Cerebellum, Neural Stem Cells, Neuroblastoma), Pancreas (Pancreatic adenocarcinoma), Pancreas (Pancreatic ductal adenocarcinoma), Pluripotent stem cell (ES cells, iPS cells), Prostate (Prostate), H3K4me1: Blood (CD4+ T cells), H3K4me1: Blood (Hematopoietic Stem Cells, Macrophages, Monocytes, Neutrophils), H3K4me1 [Liver (Hepatocytes), Pluripotent stem cell (iPS cells)], H3K4me2: Blood (CD4+ T cells, Granulocyte, Hematopoietic Stem Cells), Breast (Mammary glands), Neural (Retina), Pluripotent stem cell (iPS cells), H3K4me3: Blood (B cells, CD4+ *T*–cells, CD4 CD8 double positive cells, CD8+ T cells, Dendritic Cells, Hematopoietic Stem Cells, Macrophages, Mast Cells, Th1 Cells, Th2 Cells, Thymus), Breast (Mammary epithelial cells, Mammary glands), Liver (Hepatocytes, Liver), Lung (Lung), Neural (Neural Stem Cells), Pluripotent stem cell (ES cells, (iPS cells), Prostate (Prostate), Pluripotent stem cell (ES cells, iPS cells).

#### 5.1.3. Transcription factor data

CTCF: Blood (B cells), Blood (Erythroid Cells), Neural (Retina), kidney, lung, Pluripotent stem cell (ES cells); SPI1: Blood (Dendritic Cells), Blood (Hematopoietic Stem Cells), Blood (Macrophages), Blood (Neutrophils); TBX21: Blood (Th1 Cells); RAD21: Blood (Macrophages).

### 5.2. Data preprocessing

The preprocessing pipeline for TFBS and HM data sets prior to submitting to deep learning models is implemented as a set of shell scripts that run programs written in the Python language version 3.10. Since the size of the initial data sample is important for training of the deep learning models, we filter out experiments with the number of regions outside the range 50,000 ± 12,500 segments. The average sample size was about 40,000 segments. The selected regions were centered and extended by ±500 bp. The corresponding 1 kb sequences were extracted from corresponding genomes. We excluded regions that fall into gaps and black-listed regions.

When creating combined data sets from different tissue types, we concatenated all regions from tissue-specific experiments and merged overlapping segments.

For each original dataset we generated two additional data sets: a set of randomly selected segments in a given genome of the same size as the original sample, and a set of randomly selected segments with a size twice as large as the original sample. The first generated data set was used as a negative class during the training of deep learning model, and the second data set is used as additional data during training of the transfer learning model.

### 5.3. Baseline deep learning model

As a baseline source model, we chose hybrid CNN+LSTM architecture (Figure 3). CNN consisted of one-dimensional CNN layer with 256 kernel of size 20 followed by ReLU, Max Pooling layer of size 15, and the Dropout layer. The obtained feature representation is then directed to the bidirectional LSTM-block with a hidden layer size of 128.

**Figure 8:**
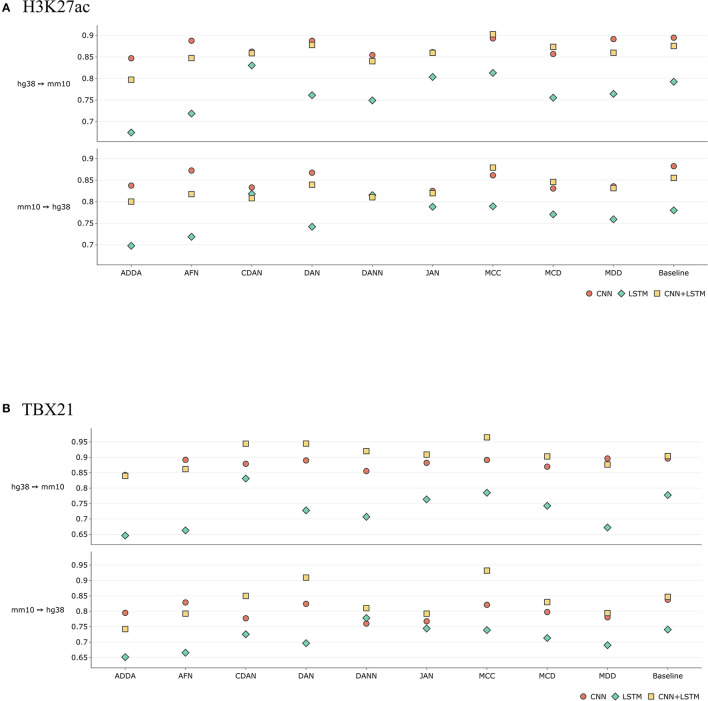
Comparative performance (ROC AUC) of Domain Adaptation methods with different baseline DL models—CNN-only, LSTM-only, and CNN+LSTM for **(A)** H3K27ac histone mark and the **(B)** TBX21 transcription factor (see comparison based on PR AUC and accuracy in Supplementary Figure 7). Y axis depicts absolute values of ROC AUC for each model. Overall, taking together ROC AUC, RP AUC, and accuracy, hybrid CNN+LSTM model outperforms CNN and LSTM models for transcription factor TBX21, and CNN outperforms CNN+LSTM for histone mark H3K27ac. When performing transfer for a particular experiment, one can make a choice between DL types based on performance of baseline model.

Figure 8 illustrates the comparison results between CNN-only, LSTM-only and CNN+LSTM network architectures on the example of H3K27ac histone mark and TBX21 transcription factor. In the first case we can see comparable performance between the CNN and the hybrid models. Interestingly, the latter considerably outperforms other methods in case of MCC. As for the overall results, the transcription factor gains are more significant both quantitatively and qualitatively.

### 5.4. DA models

For DA models implementations we adapted the scripts from https://github.com/thuml/Transfer-Learning-Library/tree/master/examples/domain_adaptation/image_classification with slight modifications to the model inputs (to provide a transition from images to nucleotide sequences).

Since the computations are time-consuming we evaluated the optimal performances achievable for DA methods by running 5 times the chosen source hybrid CNN+LSTM models with different DA approaches for two HM data sets (H3K27AC in blood and pancreas) and two transcription factors (CTCF and SPI1) (Supplementary Table 3). The results showed that DA models' performance is stable.

## 6. Computational resources

The study was performed using the supercomputer complex of the National Research University Higher School of Economics (Kostenetskiy et al., 2021).

## Data availability statement

Publicly available datasets were analyzed in this study. This data can be found here: https://chip-atlas.org. The code is freely available at https://github.com/pavellatko/dna-domain-adaptation.

## Author contributions

PL and FP performed downloaded and preprocessed the data, designed the model architectures, and wrote the scripts for automated computations on supercomputer cluster. MP and AH conceptualized and supervised the study. MP and AH wrote the manuscript with the help of FP and PL. All authors contributed to the article and approved the submitted version.
