# Supplementary material for: Unsupervised domain adaptation methods for cross-species transfer of regulatory code signals
Source: Front Big Data. 2023 Mar 30;6:1140663. doi: 10.3389/fdata.2023.1140663 (PMC10101332; doi:10.3389/fdata.2023.1140663)
Supplement: Supplementary file 3 [file Table_3.DOCX]

**Supplementary Table 3.** Cross validation of hybrid CNN+LSTM models with different DA approaches for two HM data sets (H3K27AC in blood and pancreas) and two transcription factors (CTCF and SP1)

| **H3K27AC: Blood (Macrophages)** | | | | | | |
| --- | --- | --- | --- | --- | --- | --- |
|  | **mm10 ➙ hg38** | | | **hg38 ➙ mm10** | | |
|  | **ACC** | **PR** | **ROC** | **ACC** | **PR** | **ROC** |
| **ADDA** | 70.7±0.4 | 75.4±0.2 | 74.9±0.5 | 70.8±0.5 | 81.2±0.8 | 81.0±0.2 |
| **AFN** | 72.2±0.2 | 81.0±0.3 | 80.7±0.2 | 72.4±0.2 | 82.8±0.5 | 82.3±0.4 |
| **CDAN** | 74.9±0.4 | 80.9±0.8 | 83.2±0.6 | 78.4±0.8 | 84.5±1.0 | 87.6±0.5 |
| **DAN** | 75.4±0.1 | 84.2±0.1 | 84.2±0.1 | 77.6±0.2 | 86.9±0.4 | 87.0±0.3 |
| **DANN** | 72.6±0.6 | 77.5±0.5 | 80.6±0.5 | 75.4±0.6 | 81.3±0.5 | 85.5±0.4 |
| **JAN** | 72.0±0.3 | 80.5±0.5 | 81.0±0.5 | 74.4±0.8 | 84.2±0.2 | 85.6±0.3 |
| **MCC** | 79.4±0.1 | 88.2±0.1 | 88.1±0.1 | 81.9±0.5 | 90.7±0.3 | 91.0±0.3 |
| **MCD** | 73.6±0.3 | 82.7±0.2 | 82.2±0.2 | 74.7±0.4 | 85.3±0.5 | 84.9±0.5 |
| **MDD** | 72.4±0.2 | 81.6±0.1 | 81.2±0.2 | 73.3±0.7 | 82.8±0.6 | 82.6±0.6 |
| **Source** | 74.6±0.6 | 83.8±0.4 | 83.4±0.5 | 76.2±0.3 | 85.9±0.3 | 85.6±0.3 |
|  |  |  |  |  |  |  |
| **H3K27AC: Pancreas (Pancreatic ductal adenocarcinoma)** | | | | | | |
|  | **mm10 ➙ hg38** | | | **hg38 ➙ mm10** | | |
|  | **ACC** | **PR** | **ROC** | **ACC** | **PR** | **ROC** |
| **ADDA** | 71.6±0.6 | 78.5±0.2 | 77.7±0.2 | 73.4±0.7 | 80.5±1.7 | 79.9±0.8 |
| **AFN** | 73.2±0.2 | 83.9±0.3 | 82.6±0.5 | 76.5±0.4 | 87.0±0.3 | 85.9±0.3 |
| **CDAN** | 72.7±0.0 | 78.9±1.7 | 81.6±0.4 | 77.8±0.6 | 82.3±0.4 | 86.1±0.2 |
| **DAN** | 73.5±0.4 | 85.2±0.2 | 83.9±0.2 | 78.7±0.5 | 88.8±0.2 | 88.3±0.3 |
| **DANN** | 72.2±0.2 | 78.2±0.7 | 80.9±0.3 | 75.0±0.6 | 79.2±0.7 | 84.2±0.4 |
| **JAN** | 71.6±0.2 | 82.4±0.6 | 82.0±0.2 | 74.7±0.3 | 84.1±0.8 | 85.6±0.4 |
| **MCC** | 77.9±0.3 | 88.9±0.2 | 87.7±0.3 | 81.3±0.4 | 90.9±0.1 | 90.5±0.1 |
| **MCD** | 74.9±0.2 | 86.1±0.0 | 84.6±0.1 | 78.1±0.1 | 88.1±0.0 | 87.3±0.0 |
| **MDD** | 73.1±0.1 | 85.0±0.0 | 83.2±0.1 | 76.9±0.2 | 87.0±0.2 | 86.1±0.2 |
| **Source** | 75.9±0.3 | 86.8±0.2 | 85.4±0.3 | 78.2±0.1 | 88.3±0.3 | 87.6±0.3 |
|  |  |  |  |  |  |  |
| **CTCF: Blood (B cells)** | | | | | | |
|  | **mm10 ➙ hg38** | | | **hg38 ➙ mm10** | | |
|  | **ACC** | **PR** | **ROC** | **ACC** | **PR** | **ROC** |
| **ADDA** | 63.8±0.5 | 70.1±0.2 | 69.6±0.1 | 64.2±0.4 | 68.2±0.6 | 69.2±0.8 |
| **AFN** | 65.8±1.3 | 73.0±0.7 | 72.9±1.5 | 64.6±0.3 | 69.9±0.5 | 70.4±0.8 |
| **CDAN** | 82.2±0.1 | 83.1±0.8 | 87.4±0.6 | 93.7±0.3 | 97.0±0.3 | 97.9±0.2 |
| **DAN** | 93.3±0.1 | 98.1±0.0 | 97.8±0.0 | 93.7±1.1 | 98.3±0.4 | 98.3±0.3 |
| **DANN** | 80.4±0.4 | 79.8±1.0 | 85.0±0.6 | 86.1±0.8 | 92.8±0.6 | 94.6±0.4 |
| **JAN** | 79.2±0.4 | 81.3±0.6 | 84.8±0.4 | 84.9±1.7 | 94.7±1.0 | 95.5±0.7 |
| **MCC** | 92.1±1.6 | 97.7±0.7 | 97.3±0.8 | 95.4±0.1 | 98.9±0.1 | 98.9±0.0 |
| **MCD** | 88.1±1.5 | 95.1±1.3 | 94.6±1.3 | 76.0±6.2 | 81.8±6.6 | 83.5±6.4 |
| **MDD** | 68.0±2.7 | 75.8±3.2 | 76.0±3.7 | 63.7±0.9 | 70.7±0.6 | 71.5±0.7 |
| **Source** | 89.1±2.2 | 95.6±1.3 | 95.3±1.3 | 91.6±0.8 | 96.8±0.7 | 96.9±0.5 |
|  |  |  |  |  |  |  |
| **SPI1: Blood (Dendritic Cells)** | | | | | | |
|  | **mm10 ➙ hg38** | | | **hg38 ➙ mm10** | | |
|  | **ACC** | **PR** | **ROC** | **ACC** | **PR** | **ROC** |
| **ADDA** | 57.6±0.9 | 58.1±0.9 | 60.3±1.8 | 60.8±1.1 | 63.1±3.3 | 65.2±1.8 |
| **AFN** | 58.2±0.3 | 58.7±0.2 | 61.9±0.3 | 63.0±0.8 | 67.3±1.1 | 69.5±1.8 |
| **CDAN** | 83.4±0.6 | 82.8±0.1 | 87.9±0.4 | 91.7±0.5 | 95.8±0.5 | 96.6±0.3 |
| **DAN** | 91.1±1.5 | 96.1±0.8 | 96.0±0.8 | 65.6±0.7 | 70.6±1.7 | 73.4±1.3 |
| **DANN** | 80.5±0.8 | 80.6±1.6 | 85.6±1.2 | 84.1±1.7 | 88.1±1.2 | 91.5±0.3 |
| **JAN** | 78.8±0.3 | 80.5±1.9 | 84.2±0.4 | 82.5±1.4 | 90.0±0.6 | 91.2±0.7 |
| **MCC** | 91.9±0.7 | 97.0±0.4 | 96.8±0.3 | 92.3±0.1 | 97.3±0.2 | 97.4±0.2 |
| **MCD** | 62.6±0.7 | 64.6±0.8 | 67.9±0.9 | 62.8±0.6 | 67.6±0.5 | 70.7±0.1 |
| **MDD** | 58.5±0.1 | 58.5±0.1 | 62.1±0.1 | 59.6±1.5 | 64.1±1.4 | 66.7±1.6 |
| **Source** | 81.4±1.8 | 88.4±2.0 | 88.8±1.7 | 62.7±1.1 | 69.6±0.7 | 72.5±0.8 |
