# Supplementary material for: Unsupervised domain adaptation methods for cross-species transfer of regulatory code signals
Source: Front Big Data. 2023 Mar 30;6:1140663. doi: 10.3389/fdata.2023.1140663 (PMC10101332; doi:10.3389/fdata.2023.1140663)
Supplement: Supplementary file 4 [file Data_Sheet_1.PDF]

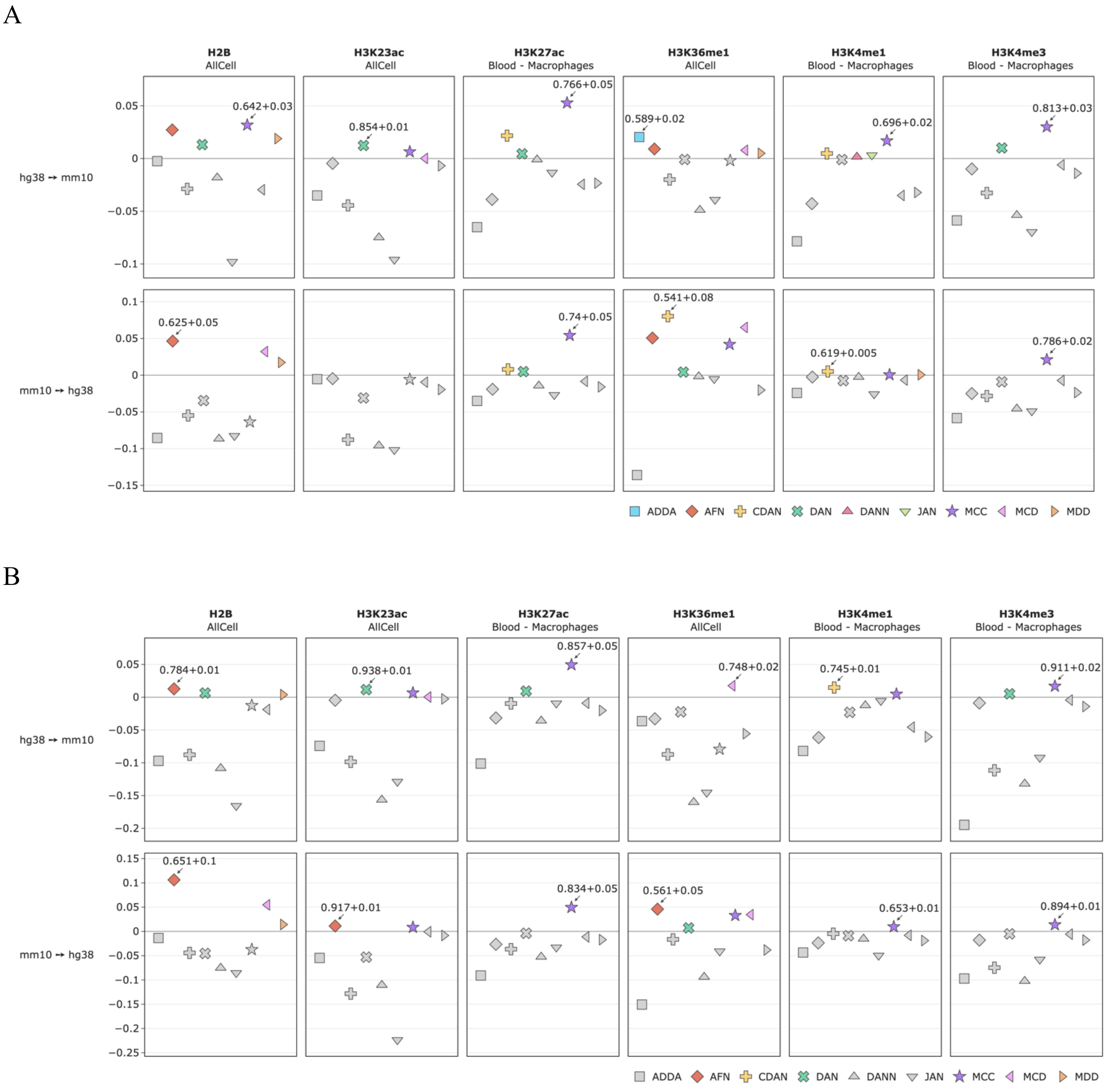

Supplementary Figure 1. Performance of Domain Adaptation methods for cross-species prediction of histone marks in terms of (A) accuracy and (B) PR AUC scores.

A

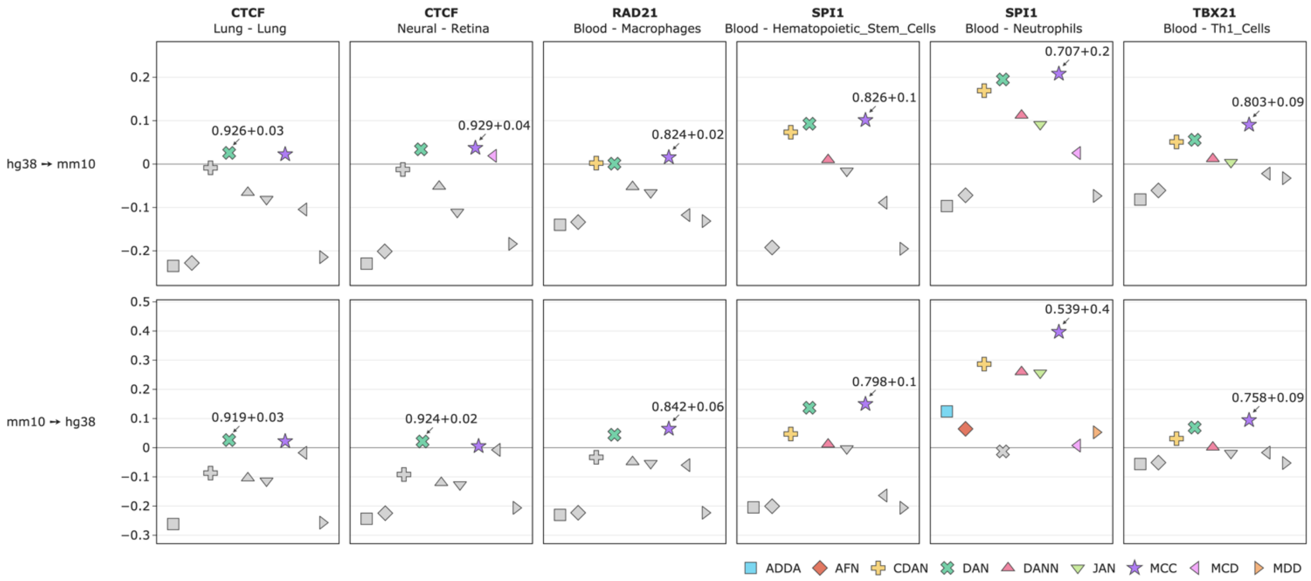

B

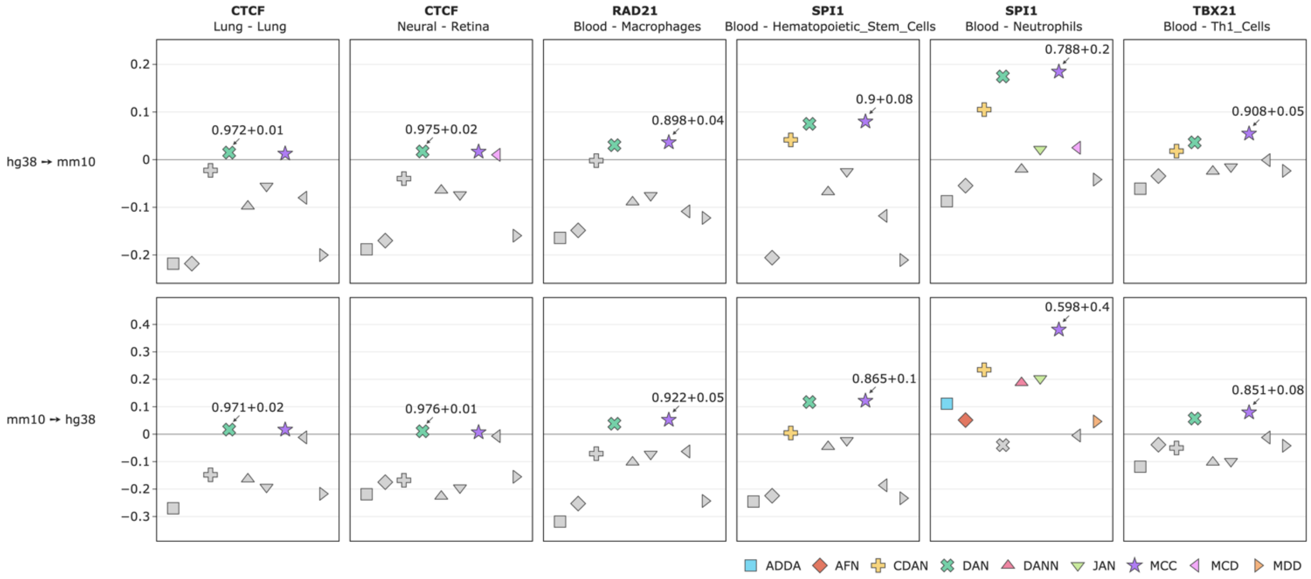

Supplementary Figure 2. Performance of Domain Adaptation methods for cross-species prediction of transcription factors in terms of (A) accuracy and (B) PR AUC scores.

A

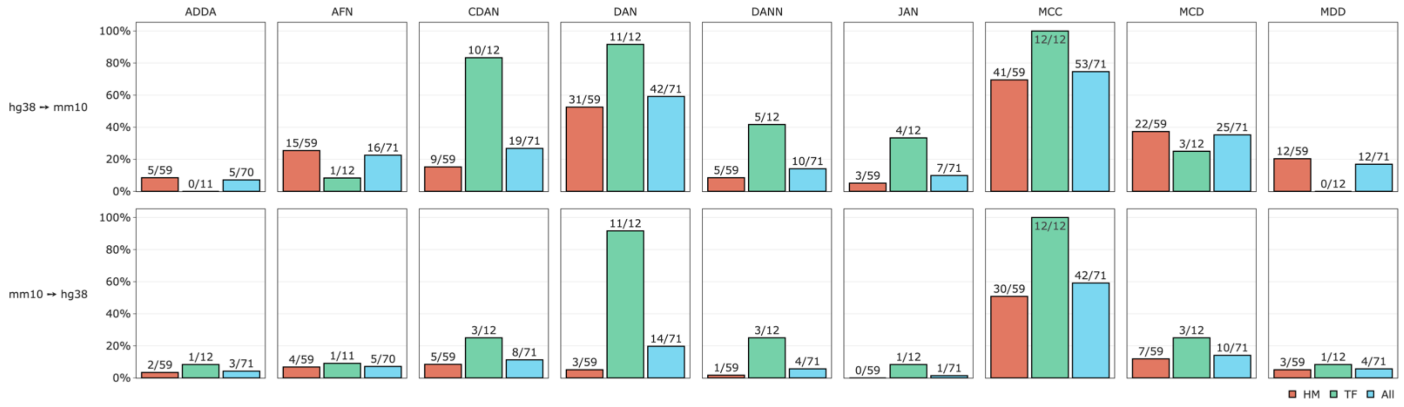

B

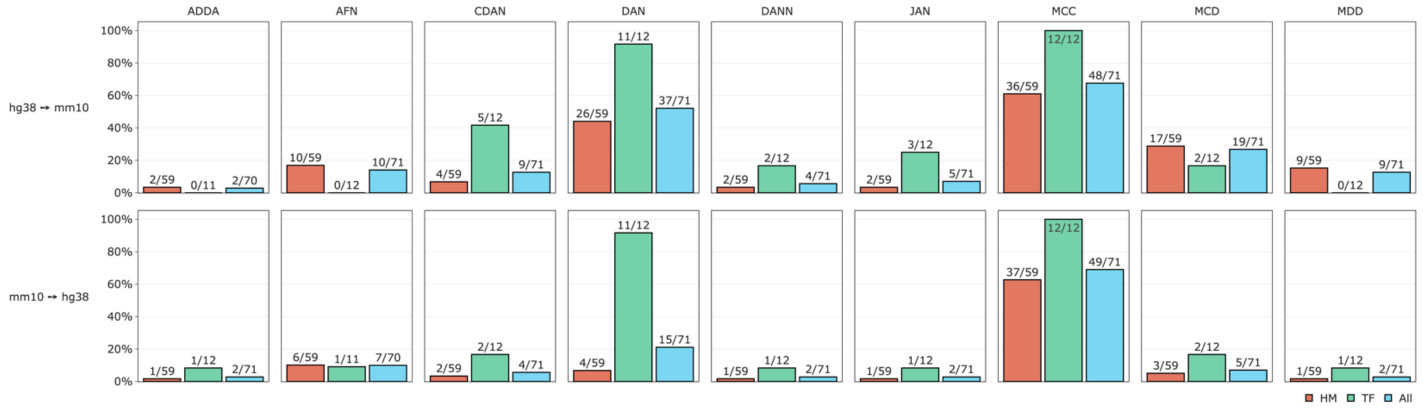

Supplementary Figure 3. Comparative performance of 9 DA models tested on TFs and HMs human-mouse and mouse-human cross-species predictions in terms of (A) accuracy and (B) PR AUC metrics.

A

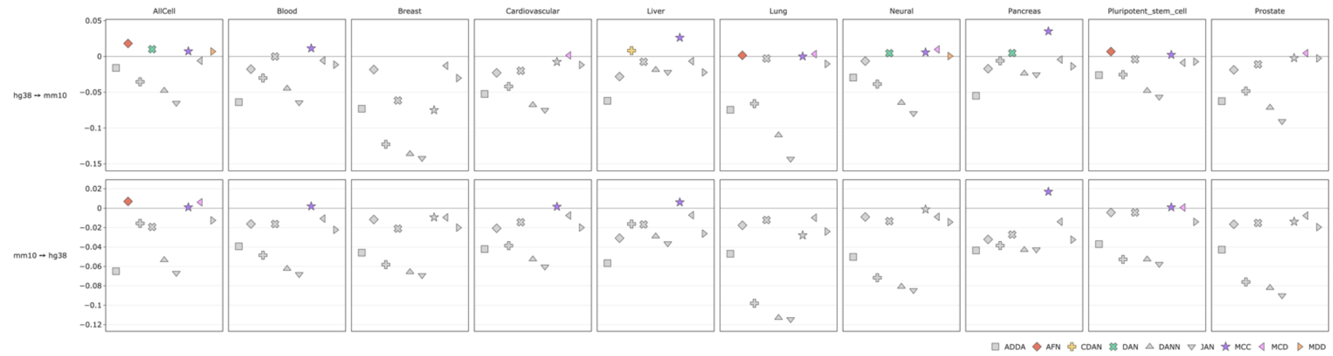

B

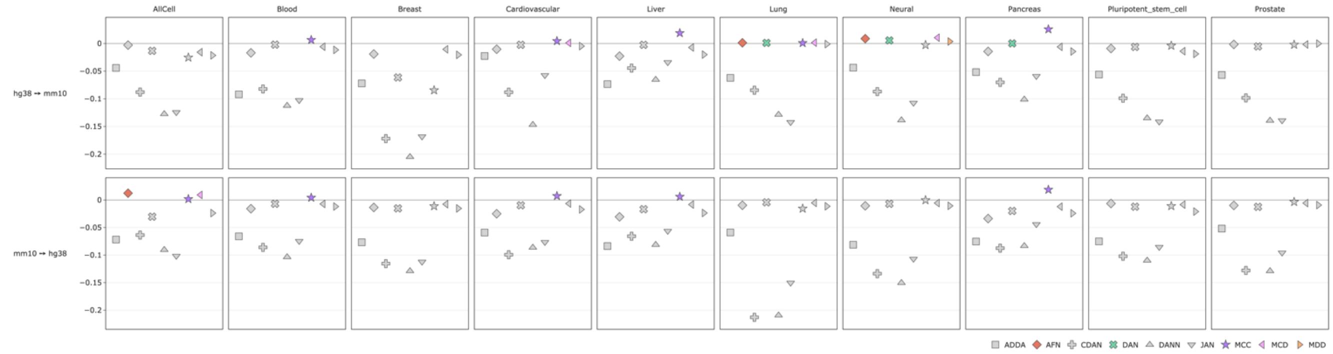

Supplementary Figure 4. Comparison of DA model performance across tissues for histone marks in terms of (A) accuracy and (B) PR AUC metrics.

A

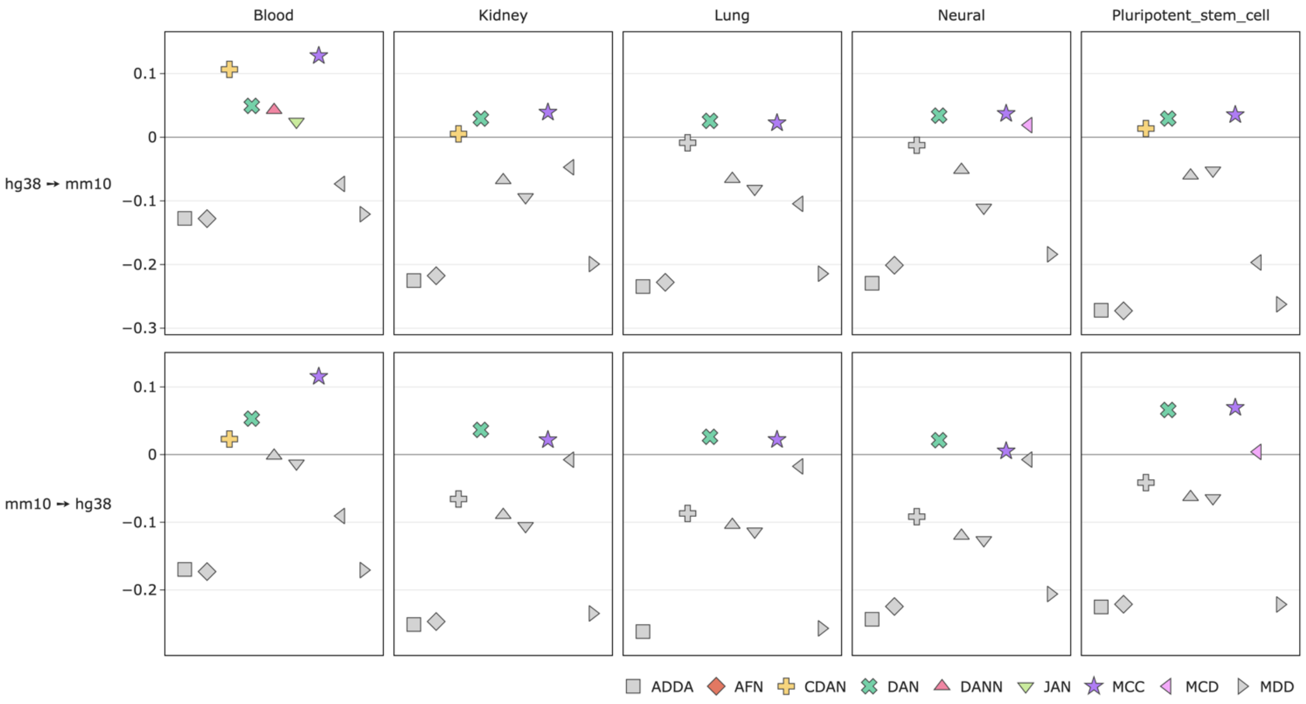

B

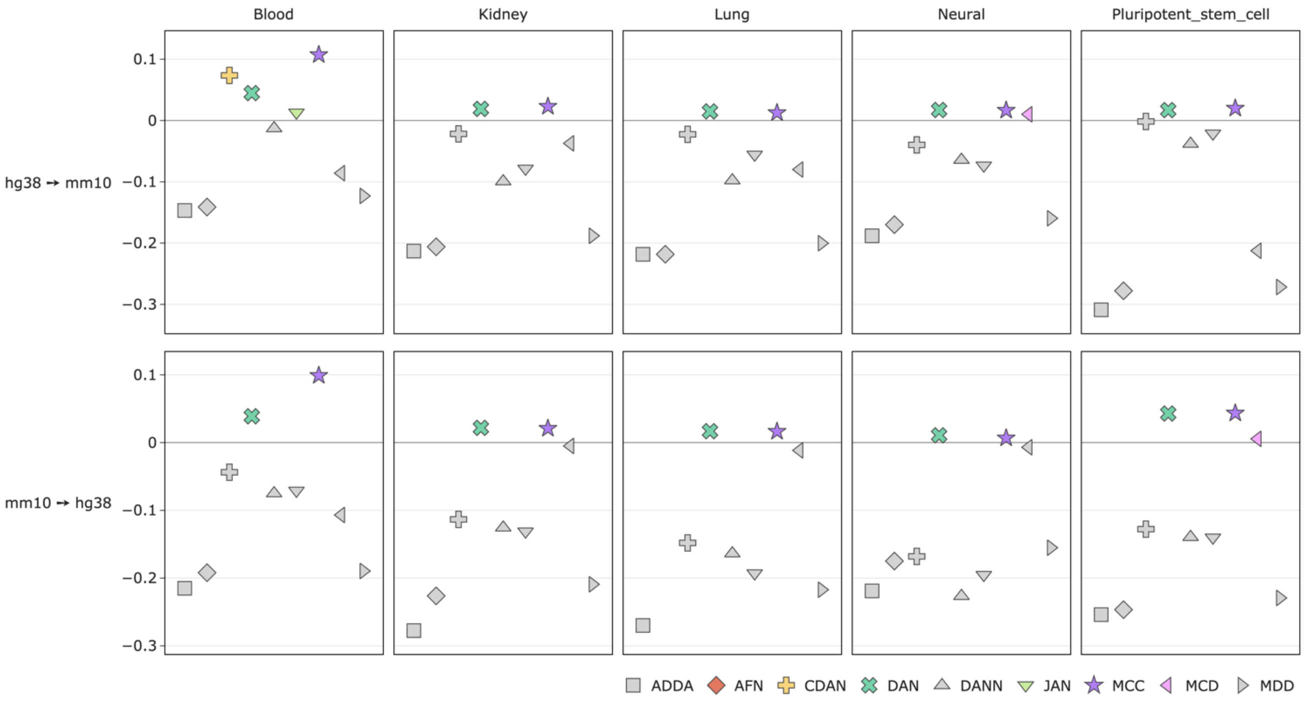

Supplementary Figure 5. Comparison of DA model performance across tissues for transcription factors in terms of (A) accuracy and (B) PR AUC metrics.

A

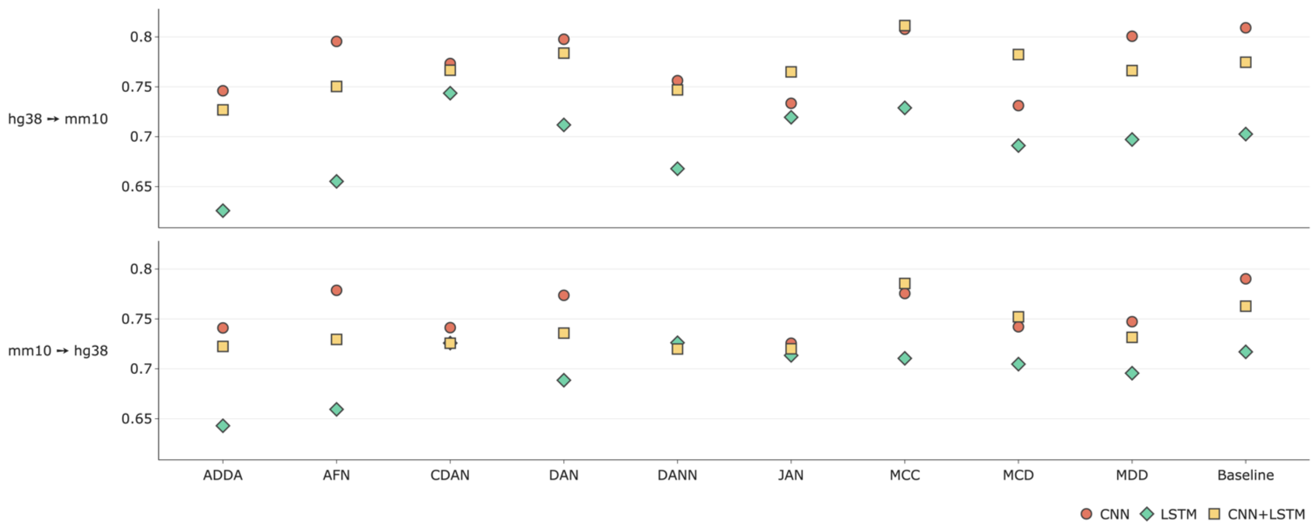

B

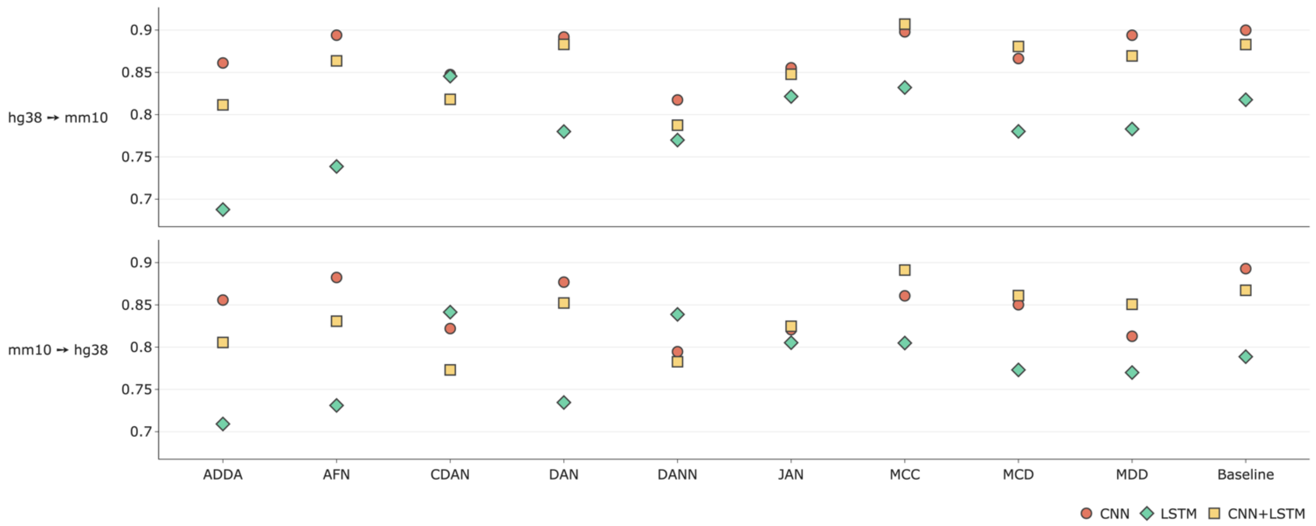

Supplementary Figure 6. Comparison of performance of different source DL models – CNN, LSTM or CNN+LSTM – used with DA approach for the H3K27ac histone mark in terms of (A) accuracy and (B) PR AUC metrics.

A

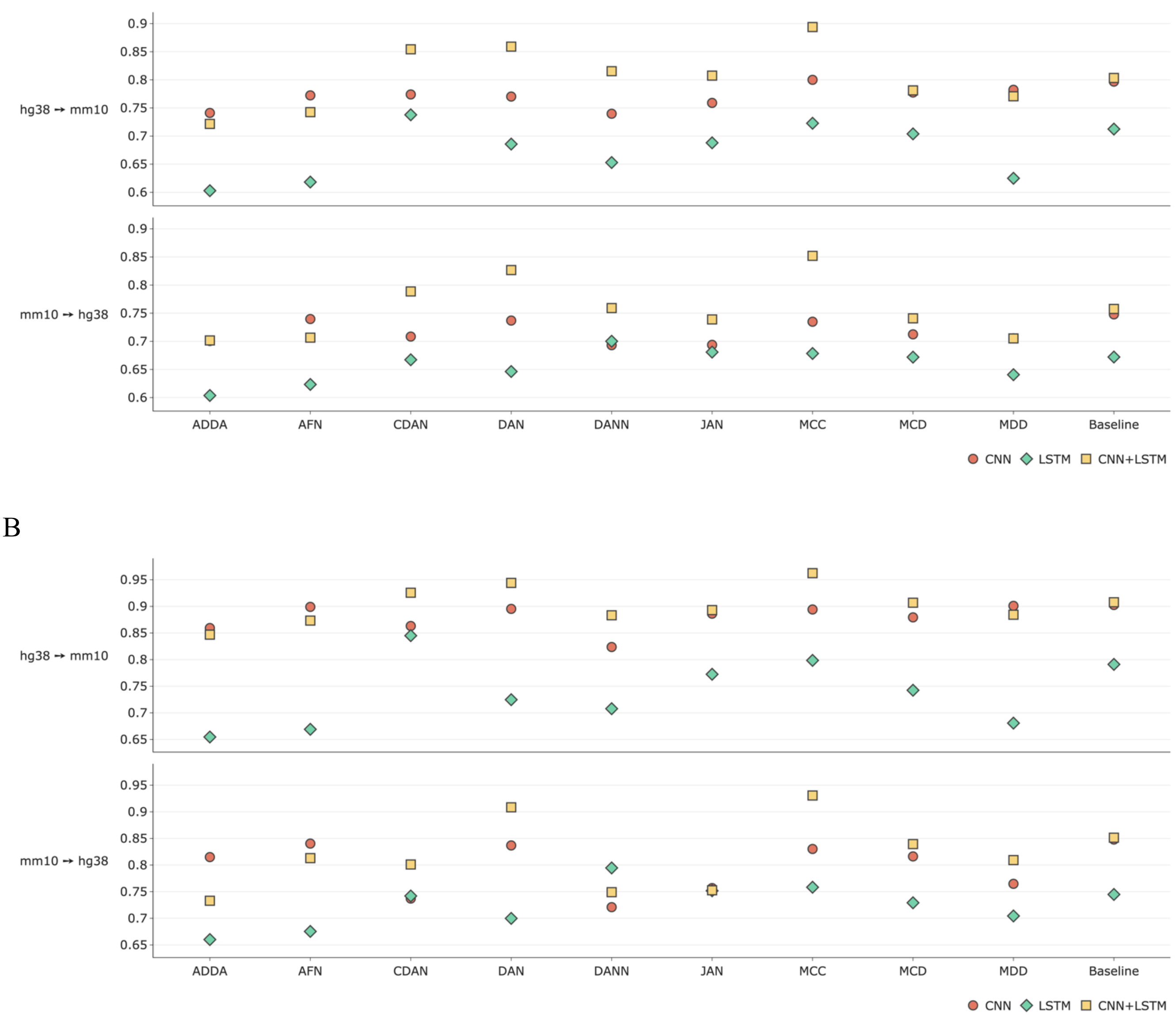

Supplementary Figure 7. Comparison of performance of different source DL models – CNN, LSTM or CNN+LSTM – used with DA approach for the TBX21 transcription factor in terms of (A) accuracy and (B) PR AUC metrics.
